# The Treatment of Bromidrosis of the Feet

**Published:** 1885-09

**Authors:** 


THE TREATMENT OF BROMIDROSIS OF THE FEET.

DR. J. S. STEWART thus writes in the *Edinburgh Medical Journal*, March, 1885:

Few affections of the skin are more disgusting or more difficult to treat successfully, by the ordinary methods, than fœtid sweating of the feet, with or without excessive secretion. The thickened skin over the heel and anterior ends of the matatarsal bones, seems to afford a very secure and ineradicable nidus for the as yet undifferentiated enzyme which induces fermentation with its fœtid products there. One form of treatment seems to be invariably successful, and therefore deserves to be much better known, viz., that devised by Hebra, which, he says, never fails, and recommends to be used in all severe cases. Hebra employed the following ointment.

℞. Olei lini,
Emplastri plumbi liquefacti, equales partes.
M. ft. ung.

This he directed to be spread thickly over a piece of linen large enough to cover the sole and sides of each foot, — both feet, in the first place, to be carefully washed and dried. Pieces of linen rag well covered with the ointment he directed to be placed between the toes, so as effectually to separate them and secure thorough application of the ointment. Over this the sock or stocking could be worn with a light slipper, and patient allowed to pursue his or her ordinary calling. This dressing to be repeated every twelve hours for ten or twelve days. The foot not to be wetted after treatment has begun, but wiped, when necessary, with a dry cloth, or washed with dry bran or other mealy substance, should any part become dirty or caked with old ointment, etc.

Whether mild or severe, all cases are curable by it, and no other method seems to yield such a prompt and satisfactory result. To insure success, the whole of the skin of soles and sides of feet and toes must be tanned by the process and gradually thrown off as brown leathery exfoliations in from two to four weeks. All boots, shoes, slippers, etc., worn by patient should be discarded; because if worn again the patient is reinfected in three or four months, and gradually becomes as bad as at first. Stockings or socks should be carefully cleansed, and disinfected by heat or by steeping in a hot solution of perchloride of mercury (1 in 1,000 of water) for several hours before being washed. Neumann directs that Hebra's ointment and dressing should be changed once in three days for nine days, that is, three times altogether, a method not to be relied on in many cases seen in this country.

What I have found to yield the most satisfactory result, in treating a tolerably long series of cases, is to have the feet thoroughly washed in hot water, then steeped for a few minutes in a solution of permanganate of potash of the strength of from four to six grains in the ounce of water. The feet are then dried, not to be again wetted until complete exfoliation of the tanned cuticle has taken place.

Hebra's lead plaster ointment is then thickly spread on strips of cloth about one and one-half inch broad, and the foot covered from the toes back over heel as high as the malleoli with these, arranged and applied like a scultetus bandage. Each toe should first be wrapped round with a strip of clean rag half an inch broad, and thickly spread with the ointment. This dressing should be renewed every twelve hours with fresh rag and ointment, for a period varying from ten to sixteen days, according to the severity of the case and the thickness of the heel skin. In most cases the odor will be very much diminished by the end of the third day, and will not be perceptible by the ninth. The shedding of the skin takes place *pari passu* with the growth of the new cuticle, and may not be completed until the end of the third or even of the fourth week.—*Quarterly Compendium of Medical Science.*

